# Long term follow-up of neoadjuvant chemotherapy for non-small cell lung cancer (NSCLC) investigating early positron emission tomography (PET) scan as a predictor of outcome

**DOI:** 10.1186/s12885-019-5284-2

**Published:** 2019-01-14

**Authors:** Perrin E. Romine, Renato G. Martins, Keith D. Eaton, Douglas E. Wood, Fatemeh Behnia, Bernardo H. L. Goulart, Michael S. Mulligan, Sarah G. Wallace, Elizabeth Kell, Julie E. Bauman, Shilpen A. Patel, Hubert J. Vesselle

**Affiliations:** 1 University of Washington, School of Medicine, 1959 NE Pacific St, Seattle, WA 98195 USA; 2 Seattle Cancer Care Alliance, 825 Eastlake Ave E, Seattle, WA 98109 USA; 3 University of Arizona, Tucson, AZ USA; 4 Grail Inc., Menlo Park, CA USA

**Keywords:** Non-small cell lung cancer, Neoadjuvant chemotherapy, (18F)-FDG PET

## Abstract

**Background:**

Neoadjuvant chemotherapy is effective in improving survival of resectable NSCLC. Based on findings in the adjuvant and metastatic setting, FDG positron emission tomography (PET) scans may offer early prognostic or predictive value after one cycle of induction chemotherapy.

**Methods:**

In this phase II non-randomized trial, patients with AJCC version 6 stage IB to IIIB operable NSCLC were treated with 3 cycles of cisplatin and pemetrexed neoadjuvant chemotherapy. Patients underwent FDG-PET scanning prior to and 18 to 21 days after the first cycle of chemotherapy. Investigators caring for patients were blinded to results, unless the scans showed evidence of disease progression. FDG-PET response was defined prospectively as a ≥ 20% decrease in the SUV of the primary lesion.

**Results:**

Between October 2005 and February 2010, 25 patients enrolled. Fifty two percent were female, 88% white, and median age was 62 years. Histology was divided into adenocarcinoma 66%, not otherwise specified (NOS) 16%, squamous cell 12%, and large cell 4%. Stage distribution was: 16% IB, 4% IIB, and 79% IIIA. Treatment was well tolerated and only one patient had a grade 4 toxicity. The median follow up was 95 months. The 5 year progression free survival (PFS) and overall survival (OS) for the entire population were 54 and 67%, respectively. Eighteen patients had a baseline FDG-PET scan and a repeat scan at day 18–21 available for comparison. Ten patients (56%) were considered metabolic responders on the day 18–21 FDG-PET scan. Responders had a 5 year PFS and OS of 60 and 70%, respectively, while the percentage for non-responders was 63 and 75% (*p* = 0.96 and 0.85).

**Conclusions:**

This phase II trial did not demonstrate that a PET scan after one cycle of chemotherapy can predict survival outcomes of patients with NSCLC treated with neoadjuvant chemotherapy.

**Trial registration:**

NCT00227539 registered September 28th, 2005.

## Background

Despite recent advances in treatment, lung cancer remains the leading cause of cancer-related death in the US and worldwide [1]. Among patients diagnosed with lung cancer, approximately 80% are diagnosed with non-small cell histology. Management of patients diagnosed with non-metastatic NSCLC includes chemotherapy, radiation therapy, and surgery. Unfortunately, among all patients who undergo surgical resection with curative intent, approximately 40% will recur [2]. Adjuvant chemotherapy has been demonstrated to improve both progression free and overall survival in resectable NSCLC [3].

Because of this, multiple studies have examined the role of neoadjuvant chemotherapy. Initial studies of neoadjuvant chemotherapy suggested a benefit in patients with mediastinal involvement [4], although long term follow up showed less of a survival benefit than originally reported [5]. Later trials demonstrated a significant 5 year overall survival advantage when using pre-operative chemotherapy in operative NSCLC, including stages IA to IIIB [6–10]. More recent data suggests that there is no difference in 3 year overall survival in patients treated with preoperative vs. postoperative chemotherapy in resectable NSCLC [3, 11, 12].

Neoadjuvant chemotherapy offers specific advantages over adjuvant treatment, including early treatment of micrometastatic disease and avoidance of treatment delays that may occur due to post-operative complications. Neoadjuvant therapy also offers the opportunity to assess chemotherapy sensitivity and response early during the treatment course and while the primary tumor is still present [13]. Various neoadjuvant regimens have been used in NSCLC, although cisplatin doublet therapy remains standard. Studies have demonstrated superior efficacy of cisplatin and pemetrexed doublet therapy compared with cisplatin and gemcitabine in patients with advanced NSCLC, excluding patients with squamous cell histology. Pemetrexed based regimens are better tolerated than gemcitabine based regimens, motivating our selection of this regimen for patients with adenocarcinoma [14].

Ideally, molecular markers would be used to define early chemotherapy sensitivity and response. However, such markers are not yet available for lung cancer. Alternatively, FDG-PET imaging could be used as a physiological test of response to chemotherapy. FDG-PET is the standard of care in initial staging of NSCLC and in guiding its surgical staging [15, 16]. Sequential FDG-PET imaging has been evaluated as a metric for response to treatment in NSCLC [17–19]. Studies have indicated that FDG-PET can be used to differentiate radiologic responders from non-responders following one cycle of cisplatin based neoadjuvant chemotherapy [20] and histopathologic response during neoadjuvant erlotinib therapy [19, 21] in stage IB-IIIA NSCLC. Chaft et al. published results from a phase II trial of resectable NSCLC patients receiving neoadjuvant chemotherapy with FDG-PET imaging done following two cycles of cisplatin based therapy. The chemotherapy regimen was maintained or changed based on initial metabolic response following two cycles of treatment. This study demonstrated an improved radiographic response by altering treatment based on interval imaging but was not designed to evaluate for survival [22].

Despite increasing research on the use of FDG-PET imaging in NSCLC, studies have failed to consistently demonstrate a predicted survival benefit based on FDG-PET imaging results following neoadjuvant chemotherapy [23–30]. Furthermore, these studies fail to address the potential benefit of early quantitative response information as measured by FDG-PET imaging during neoadjuvant treatment. In this phase II study, we aim to test if FDG-PET response early during neoadjuvant chemotherapy predicts for a survival benefit in resectable NSCLC.

## Materials and methods

### Patient population

This study was conducted with the approval and supervision of the University of Washington Institutional Review Board (IRB) and was registered at clinicaltrials.gov (NCT 00227539.) Eligible patients were defined as having histological or cytological documented NSCLC who were deemed to be candidates for surgical resection with clinical stages IB-IIIB (T4, N0–2) disease. Timing of mediastinal staging with mediastinoscopy (pre-neoadjuvant chemotherapy or at the time of surgery) was decided at the discretion of the surgeon. Patients were required to have an FDG-PET scan with measurable disease as part of initial staging, defined according to RECIST 1.0 criteria [31]. Patients were required to have adequate pulmonary reserve with a predicted FEV1 > 0.8 L following proposed resection. Patients were also required to have adequate organ function, defined as bilirubin ≤1.5xULN, AST and ALT ≤3.0xULN, CrCl ≥45 ml/min, platelet count ≥100 × 10^9^/L, and ANC ≥ 1.25 × 10^9^/L. All patients were age ≥ 18 with ECOG performance status of 0 or 1. Patients were excluded if they had been treated with any non-FDA approved or investigational drugs within 30 days of enrolling, had unresectable disease or a second primary malignancy, had a malignant pleural effusion, had prior radiation therapy to the chest, had type 1 diabetes (type 2 diabetes patients were included if glucose levels were well controlled), were pregnant or nursing, were unable to interrupt aspirin or NSAID therapy for 5 days, or if they were unable to take any of the required study drugs, including corticosteroids, folic acid, or vitamin B12. All patients provided informed consent. In total, 25 patients were recruited to this phase II study.

### Preoperative treatment

All patients were treated with a maximum of three cycles of neoadjuvant cisplatin 75 mg/m^2^ and pemetrexed 500 mg/m^2^ on day 1 of a 21 day cycle. Following the phase III trial by Scagliotti et al. demonstrating inferior survival for squamous cell patients treated with cisplatin/pemetrexed, patients with squamous cell pathology were excluded [14]. Interval FDG-PET scans were not used to make treatment decisions, except when scans showed disease progression precluding surgical cure, in which case patients were withdrawn from the study.

### Radiologic analysis

The initial FDG-PET scan was done within 4 weeks preceding the first cycle of neoadjuvant chemotherapy with concomitant CT scan of the chest and upper abdomen. Patients were considered eligible regardless of the performing location of their initial FDG-PET and CT scan. Repeat FDG-PET scans were performed at the Seattle Cancer Care Alliance/University of Washington Medical Center after the first (between day 18 and 21) and third cycle (day 64) of neoadjuvant chemotherapy. Repeat CT of the chest and abdomen was performed after the third cycle of chemotherapy prior to surgery. All PET imaging done at SCCA/UWMC was performed on a GE Discovery DSTE PET/CT system (GE Medical Systems, Waukesha, WI).

Prior to FDG-PET imaging, patients fasted for 12 h. Immediately prior to imaging, patients were confirmed to have blood glucose measurements between 80 and 150 mg/dl. Patients received an IV injection of 10 mCi F-18 FDG followed by a 60-min period of rest. An initial emission scan was done over 5 fields-of-view of 7 min duration each, encompassing the head, neck, thorax, abdomen, and pelvis. A 2.5 mm non-contrast axial CT scan was performed for localization and attenuation correction. Target lesions on CT imaging were selected on the basis of size (longest diameter) and their suitability for accurate repeated measurements.

### Quantitative analysis

FDG-PET standardized uptake value (SUV) was evaluated in the primary tumor of each subject. A SUV was defined as the time averaged tissue activity C (μCi/ml), from 60 to 67 min following injection, divided by the injected dose ID (mCi) per kilogram of patient body weight. The maximum SUV value (SUVmax) was used for the primary tumor response, defined as the maximum pixel value within a region of interest (ROI) encompassing the entire primary lesion on the SUV images. Percentage SUV changes between initial and subsequent FDG-PET scans were calculated based on primary tumor SUVmax.

### Statistical analysis

The primary objective of this trial was to evaluate the potential effectiveness of FDG-PET after one cycle of chemotherapy in predicting radiographic response to neo-adjuvant chemotherapy in patients with operable stage IB to IIIB NSCLC. Previous work in a patient population with advanced disease indicated that a decrease in tumor SUV of 20% or greater was associated with radiographic response with a PPV of 0.71 and a NPV of 0.96 [27]. A sample size of 35 was chosen to provide sufficient power to evaluate different FDG-PET response thresholds under varying assumptions of response rate by CT. A lower than predicted CT response rate observed in this study precluded analysis of different thresholds and we therefore analyzed the data based on the 20% threshold. Secondary outcomes were PFS and OS.

All patients were followed at 3-month intervals for the initial 2 years, then at 6-month intervals for a subsequent 3 years. A 20% decrease in SUV of the primary tumor as measured by FDG-PET was utilized as the threshold for metabolic response, given prior work by Weber et al. demonstrating that this was associated with radiographic response [27]. Radiographic response was defined per RECIST 1.0 criteria [31]. Complete response was defined as complete resolution of all radiological abnormalities. Partial response was defined as at least a 30% decrease in the sum of the longest diameter of the target lesions. Progressive disease was defined as at least a 20% increase in the sum of the longest diameter of the target lesion. Stable disease was defined in patients that did not qualify as partial response or progressive disease. *P* values less than 0.05 were considered significant. PFS and OS were assessed using the Kaplan-Meier log-rank method. Post-hoc analysis was performed further evaluating survival trends based on metabolic response on post-chemotherapy FDG-PET, excluding patients with squamous cell histology, and utilizing a 30% decrease in SUV as the threshold for metabolic response per PERCIST criteria [32].

## Results

### Patient characteristics

**Fig. 1:**
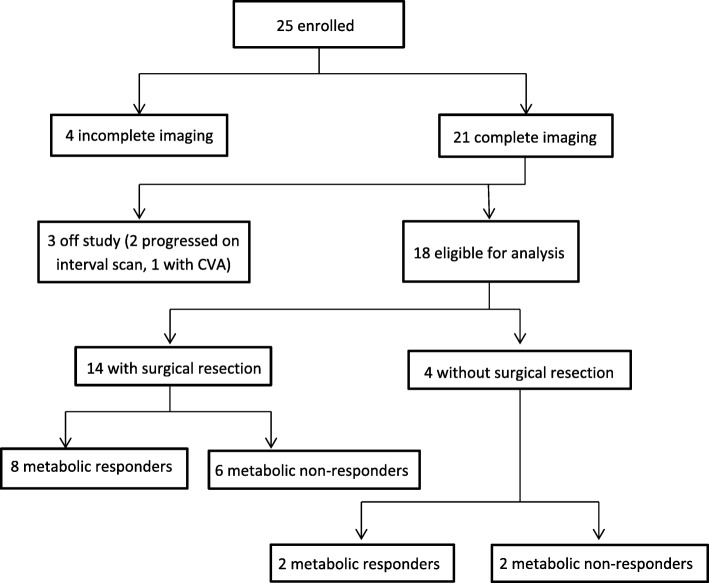
CONSORT flow diagram; CVA, cerebral vascular accident

Twenty-five patients were enrolled between October 2005 and February 2010. Due to slow recruitment, this study was closed prior to enrolling the goal sample size. Baseline patient characteristics are summarized in Table 1. One patient was excluded from analysis following surgical resection due to reclassification of her tumor histology as carcinoid (despite two independent reviews indicating NSCLC in the initial biopsy). Fifty percent of patients underwent mediastinal staging prior to neoadjuvant chemotherapy; the remainder of patients underwent mediastinal staging following neoadjuvant chemotherapy. Of the 19 patients with Stage IIIA disease, 11 (58%) had confirmed pathologic N2 disease prior to neoadjuvant treatment. Of the remaining eight patients, three had confirmed N2 disease following neoadjuvant treatment while five had confirmed N0-N1 disease. Treatment was well tolerated. One patient had a grade 4 toxicity involving a cerebral vascular accident (CVA); this patient was subsequently withdrawn from the study. Nine patients had grade three toxicities, of which six were felt to be directly related to the study treatment (Table 2). Three patients withdrew from the study: one due to the previously mentioned CVA, two due to disease progression while on neoadjuvant treatment. In total, 18 patients were eligible for final analysis, of which 14 underwent definitive surgical resection (Fig. 1). Four patients did not undergo surgical resection due to disease progression following neoadjuvant chemotherapy.

**Table 1:** Patient Characteristics

Characteristic	Number
Female sex, n (%)	13 (52)
Median age, years	62
Histologic Subtype, n (%)	
Adenocarcinoma	16 (67)
Squamous cell carcinoma	3 (13)
NSCLC, not otherwise specified	4 (16)
Large cell carcinoma	1 (4)
Clinical stage, n (%)	
IB	4 (16)
IIA	0
IIB	1 (4)
IIIA	19 (79)
Smoking, n (%)	
Yes	23 (92)
No	2 (8)
PS, n (%)	
0	19 (76)
1	4 (16)

*NSCLC* non-small cell lung cancer, *PS* performance status

**Table 2:** Adverse Events

Toxicity Category	Grade	Number (%)
Fatigue	3	7 (28)
Pain	3	2 (8)
Metabolic/Laboratory	3	1 (4)
Pulmonary	3	3 (12)
Gastrointestinal	3	4 (16)
Dermatologic/skin	3	2 (8)
Neurologic	4	1 (4)
Allergy/Immunologic	3	1 (4)

Note: Maximum grade per patient per body system presented. Number of evaluable patients: 25. Total number of patients with grade 3/4 toxicities: 10 (patients with multiple toxicities and are listed separately above)

### Radiologic and pathologic response

Of the eighteen evaluable patients, 14 (78%) had stable disease on repeat chest CT following 3 cycles of neoadjuvant therapy, 2 (11%) had a partial response, and one (6%) had progressive disease. One patient did not have baseline chest CT images available for comparison. None of the fourteen patients who underwent definitive surgical resection had a pathologic complete response following 3 cycles of neoadjuvant chemotherapy.

### Metabolic response

Ten patients (56%) were considered metabolic responders on day 18–21 FDG-PET, using a 20% reduction in SUV as a threshold. Nine patients (50%) were considered metabolic responders on day 18–21 FDG-PET when the SUV threshold was adjusted to 30%. There was a median 28.9% decrease in interval SUV, ranging from an 80.5% decrease to a 46.3% increase. In total, 17 patients had complete FDG-PET and CT imaging. Of the 10 patients considered early metabolic responders, 8 had stable disease on repeat chest CT following 3 cycles of neoadjuvant therapy while 2 had a partial response. Of the 7 patients considered metabolic non-responders, 6 had stable disease on repeat CT imaging while 1 had progressive disease. Among the 4 patients with disease progression following neoadjuvant chemotherapy, 2 were deemed early metabolic responders.

### Progression free survival and overall survival

**Fig. 2:**
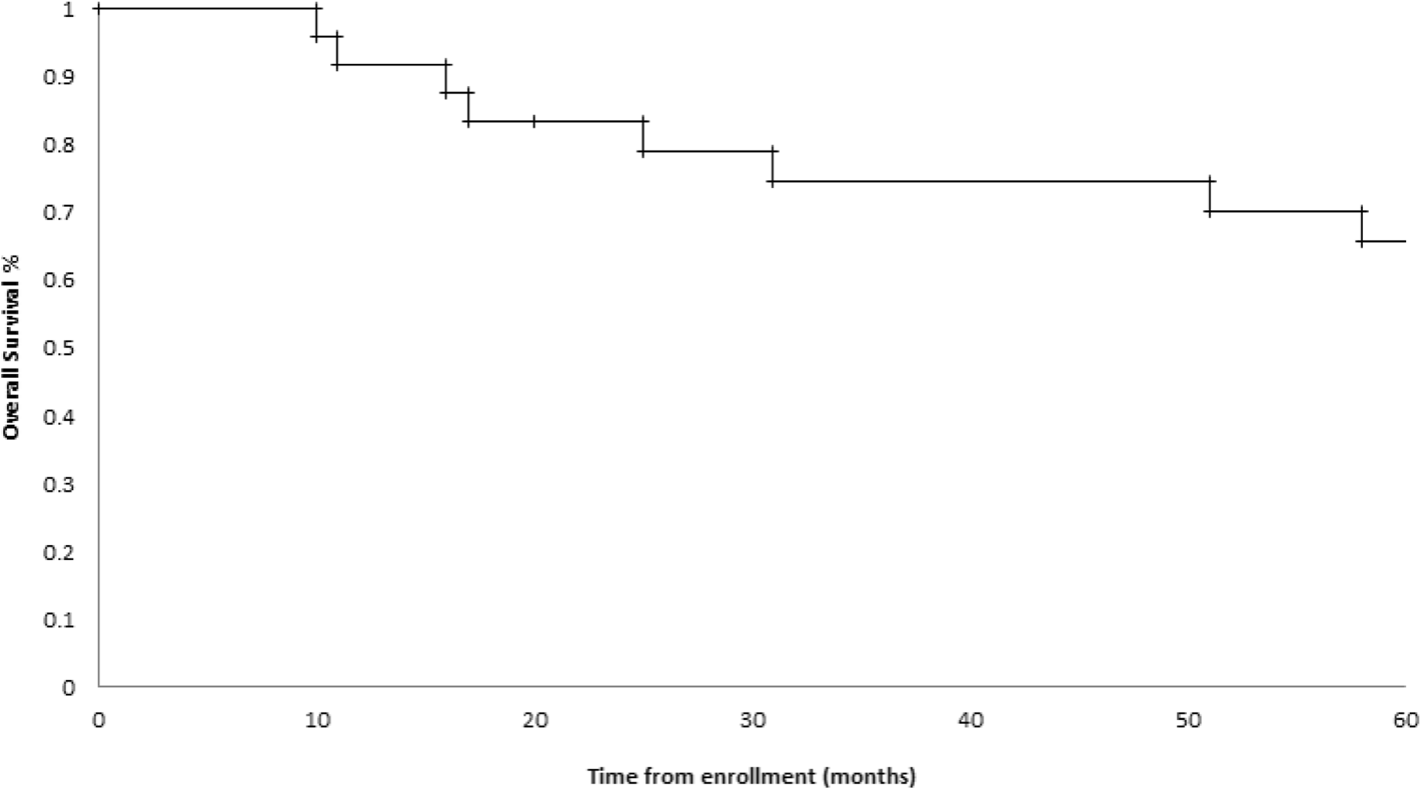
Overall Survival of the entire cohort censored at 5 years

**Fig. 3:**
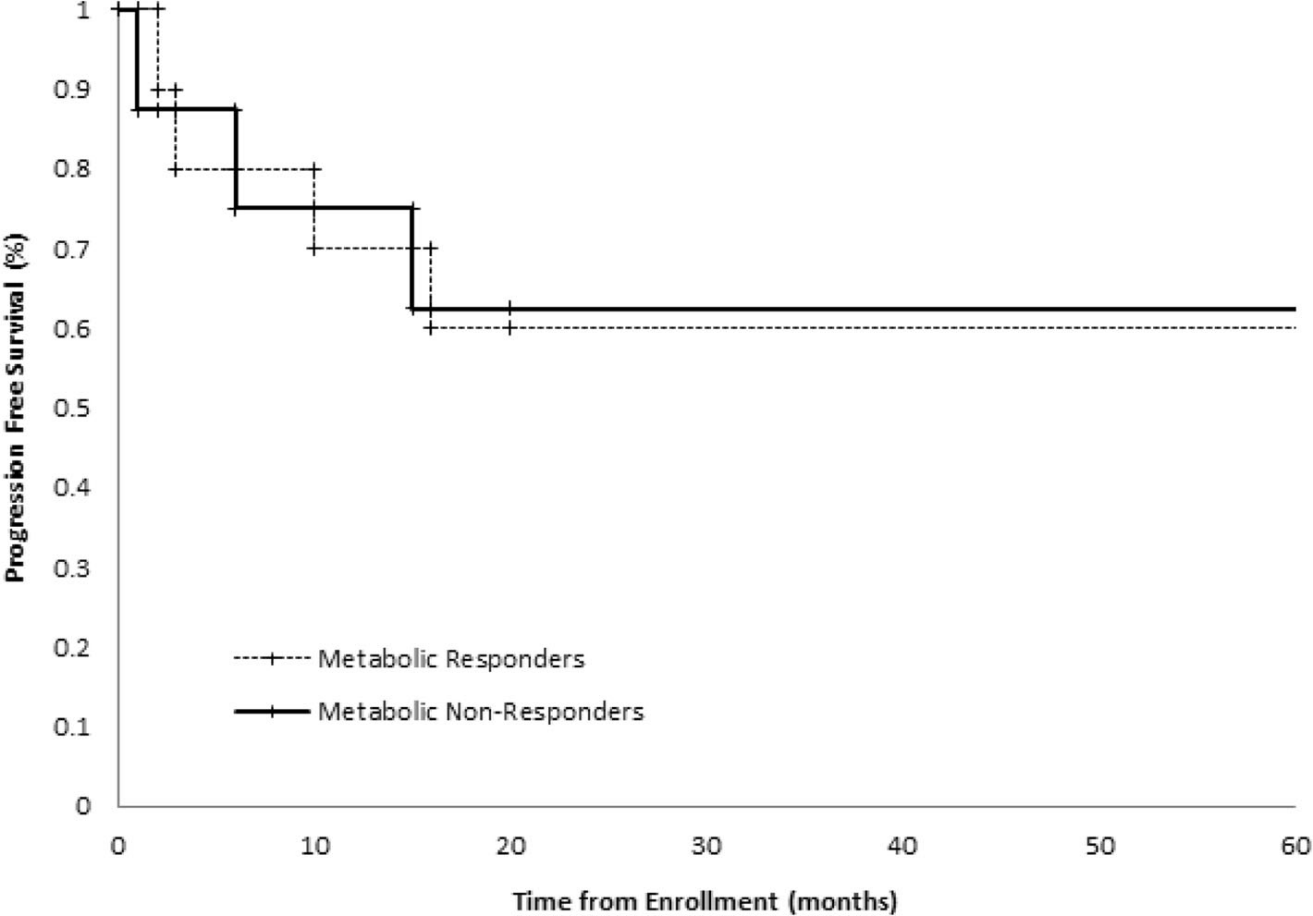
Kaplan-Meier progression free survival curve, stratified by metabolic response on interval FDGPET imaging. Response was defined as ≥20% decrease in SUV of the dominant lesion

**Fig. 4:**
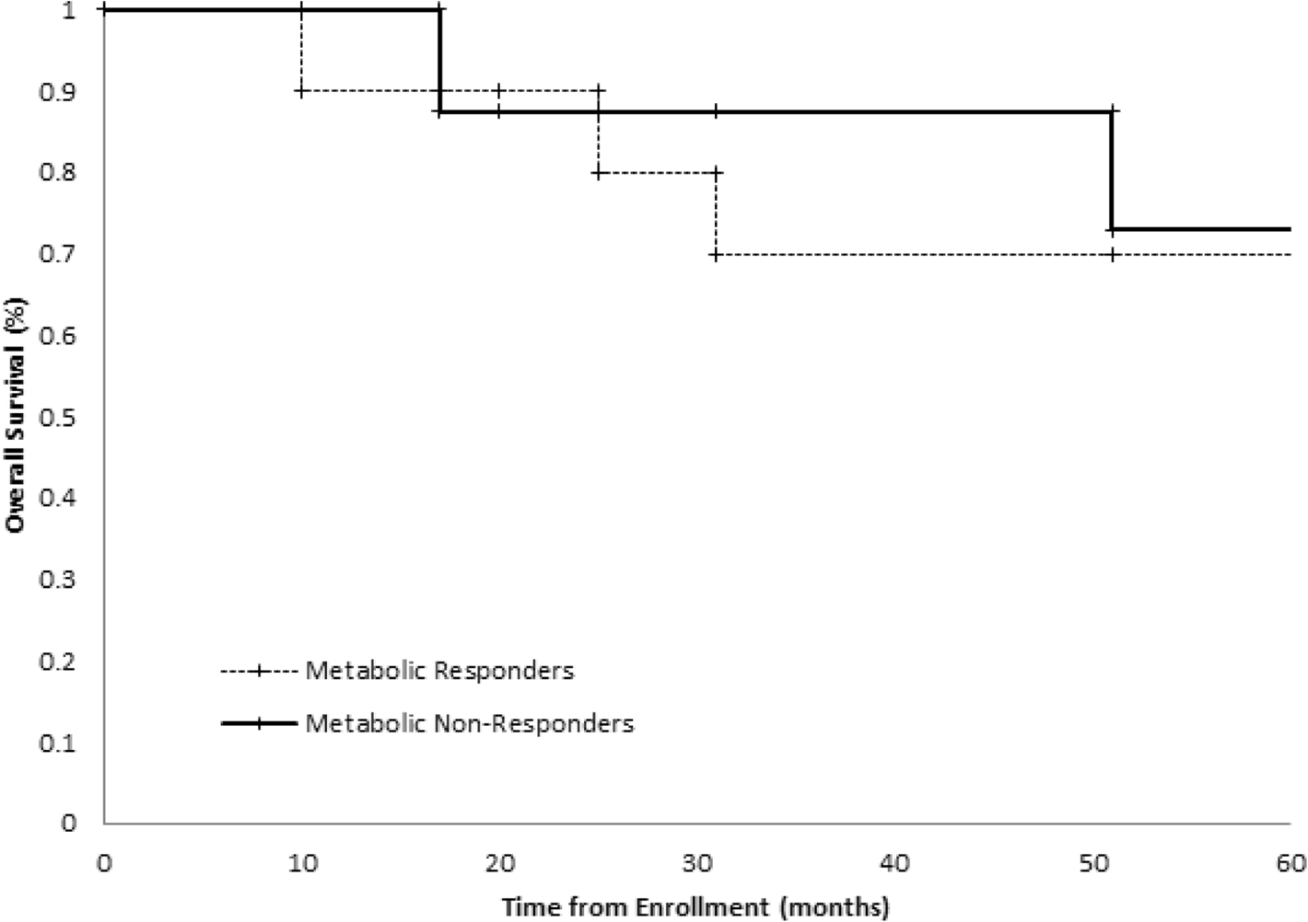
Kaplan-Meier overall survival curve, stratified by radiologic response on interval FDG-PET imaging. Response was defined as ≥20% decrease in SUV of the dominant lesion

The median followup of study participants was 95 months. The 5 year PFS and OS for the entire population were 54 and 67%, respectively. The overall survival curve for the entire cohort is shown in Fig. 2. When stratified by metabolic response, PFS at 5 years for responders and non-responders was 60 and 63%, respectively. OS at 5 years for responders was 70%, while the percentage for non-responders was 75%. Kaplan-Meier curves for PFS and OS are shown in Figs. 3 and 4 (*p*-values 0.96 and 0.85, respectively). In an intention to treat analysis, PFS and OS of non-responders decreased to 50 and 64% at 5 years, respectively, with no change in Kaplan-Meier log-rank statistics (p-value 0.62 and 0.61). PFS and OS trends were re-analyzed in a post-hoc analysis excluding patients with squamous cell histology (p-value 0.91 and 0.89, respectively) and using a 30% decrease in SUV as the threshold for metabolic response (p-value 0.65 and 0.33, respectively). PFS and OS trends were further re-analyzed utilizing metabolic response following completion of neoadjuvant chemotherapy (also utilizing a 20% reduction in SUV as a threshold for metabolic response). There was no significant difference in either survival trend (*p*-value 0.55 and 0.50, respectively).

## Discussion

FDG-PET imaging has the potential to provide early quantitative response information during neoadjuvant treatment of NSCLC. Our aim was to evaluate imaging done following one cycle of neoadjuvant treatment as a means of measuring early prognostic information in NSCLC. Studies have shown that FDG-PET imaging during neoadjuvant treatment (after one or two cycles) is predictive of later radiographic response, and can possibly be used to adapt chemotherapy regimen [20, 22]. Hoekstra et al. demonstrate that metabolic response on FDG-PET imaging done after one cycle of platinum-based neoadjuvant chemotherapy is predictive of prognosis [26]. Contrary to this, our study did not demonstrate a clear association between early PET assessment with later CT response. No change in survival was observed when patients with squamous cell histology were excluded from the analysis or when the threshold for metabolic response was adjusted to 30%.

There are multiple factors that may have influenced our results. This study is underpowered to show potential clinically significant survival differences. However, the survival curves shown in Figs. 3 and 4 overlap, suggesting that a significant survival difference is unlikely. Five-year overall survival of the accrued cohort was 67%. This remains higher than expected based on reported large scale meta-analyses of NSCLC, even when accounting for the predominance of patients with Stage IIIA disease in our cohort and the expected survival benefit from neoadjuvant chemotherapy [7, 33]. While this likely arose by chance and reflects the small size of our cohort and the single institutional nature of this trial, this disparity may impact the predictive nature of early FDG-PET imaging within this study cohort.

In addition, this trial included patients with initial FDG-PET imaging done at other institutions. Test-retest repeatability studies of multicenter FDG-PET scans have demonstrated that a decrease in SUVmax by 30% in scans done at the same institution is unlikely to reflect variability in measurement [34]. However, our study design, while representative of clinical practice, could lead to further variability in SUV quantifications.

As this study illustrates, the utility of early FDG-PET scanning during neo-adjuvant therapy, optimal timing, and the criteria for imaging response are areas that require further research. By repeating imaging following only one cycle of neoadjuvant chemotherapy, we hoped to demonstrate early predictive information that could be used to drive treatment choices and reduce unnecessary treatment related morbidity. Prior work following patients with inoperable NSCLC with weekly FDG-PET scans during the first 7 weeks of chemotherapy has demonstrated that prognostic information can be determined as early as 3 weeks [35]. However, it remains unclear if these results can be extrapolated to patients with operable NSCLC receiving neoadjuvant therapy.

Numerous methods are currently used to assess tumor response as measured by FDG-PET imaging. By defining metabolic response prospectively, rather than post-hoc, we hoped to avoid bias in interpreting FDG-PET response. SUV is widely used in the clinical setting across practice settings and does not require dynamic data acquisition or complex data analysis. A response based on SUV change, therefore, offers significant advantages when considering clinical implementation. A 20% threshold was chosen based on prior work demonstrating 10% reproducibility in SUV measurements; by using 20% we aimed to capture patients with a change in SUV that was greater that two times the standard deviation [27]. However, it is possible that a more stringent definition of metabolic response, such as the dynamic metabolic rate of glucose used by Hoekstra et al., is necessary to accurately predict prognostic information [26].

Multiple studies have looked at the predictive value of FDG-PET following, rather than during, neoadjuvant treatment of NSCLC. Results from these studies have been variable, with some studies demonstrating an association between metabolic response and survival, while others fail to demonstrate a predicted survival benefit [23–25, 28–30]. While the results are disparate, it should be noted that study design, choice of neoadjuvant therapy, cohort size, and clinical stage of included patients varied greatly between these studies. In particular, FDG-PET metabolic response definitions are highly inconsistent, ranging from a significant decrease in the metabolism of the index lesion by direct comparison as determined by a reading nuclear radiologist [29] to absolute SUV cutoffs on follow up imaging [23, 25] to variable rates of SUV change on follow up imaging [24, 28, 30]. These inconsistencies may in part explain the variable results seen across these studies. Our study, while designed to evaluate early FDG-PET response, did not demonstrate a predicted survival benefit when analyzing FDG-PET imaging done following completion of neoadjuvant therapy.

In conclusion, this study failed to demonstrate that early FDG-PET imaging after one cycle of neoadjuvant chemotherapy can predict survival outcomes of patients with NSCLC. As utilization of neoadjuvant treatment changes in NSCLC patients, further work is needed to better understand the optimal timing and method to assess early chemotherapy sensitivity and response.

## Abbreviations

AJCC: American Joint Committee on Cancer
ALT: Alanine aminotransferase
ANC: Absolute neutrophil count
AST: Aspartate aminotransferase
CrCL: Creatinine clearance
CT: Computed tomography
CVA: Cerebral vascular accident
ECOG: Eastern cooperative oncology group
FDA: Food and drug administration
FDG: Fluorodeoxyglucose
FEV: Forced expiratory volume
ID: Injected dose
IRB: Institutional review board
NOS: Not otherwise specified
NSAID: Non-steroidal anti-inflammatory drug
NSCLC: Non-small cell lung cancer
OS: Overall survival
PERCIST: Positron emission tomography response criteria in solid tumors
PET: Positron emission tomography
PFS: Progression free survival
RECIST: Response evaluation criteria in solid tumors
ROI: Region of interest
SUV: Standardized uptake value
ULN: Upper limit of normal
